# The Link Between Depression, Analgesia Usage and Function in Osteoarthritis: A Propensity Score-Matched Analysis from the Osteoarthritis Initiative Cohort

**DOI:** 10.3390/bioengineering13010063

**Published:** 2026-01-06

**Authors:** Saran Singh Gill, Gareth G. Jones, Justin P. Cobb, M. Abdulhadi Alagha

**Affiliations:** MSk Lab, Department of Surgery and Cancer, Faculty of Medicine, Imperial College London, London W12 0BZ, UK

**Keywords:** depression, knee osteoarthritis, biopsychosocial model

## Abstract

Knee osteoarthritis (OA) affects around 37% of U.S. adults over 60, with over 25% experience depressive symptoms (DSs), linked to worse pain and outcomes. Yet their impact on analgesic use and recovery remains unclear. This study aimed to assess if DSs influence analgesic use and functional outcomes in knee OA. Using data from the Osteoarthritis Initiative (n = 3680), we used a Machine Learning (ML)-based Gradient Boosting Machine (GBM) model to perform propensity score matching, matching patients with knee OA and DSs (n = 487) to those without DSs (n = 487). Outcomes at baseline, 1 and 2 years included analgesic use, function (WOMAC), quality of life (KOOS-QoL), and physical health (SF-12 PCS). Regression and timepoint models compared follow-up with baseline. DSs alone were not associated with greater opioid use up to Year 2 (OR = 0.89, 95% CI: 0.45–1.73; *p* = 0.73). Among patients with DSs, SF-12 PCS improvement was less likely at Year 1, while decline was more likely up to Year 2. DSs in OA were linked to poorer physical health, but often greater functional gains than those in OA without DSs, with no difference in opioid use. These findings highlight the need for multidisciplinary strategies, addressing both pain and psychosocial wellbeing.

## 1. Introduction

Knee osteoarthritis (OA) is the most prevalent joint disease worldwide and a leading contributor to global disability, affecting an estimated 37% of individuals over 60 years of age in the United States (US), a figure forecasted to rise to over 50% in the coming years [1,2,3,4,5]. Depressive symptoms are prevalent among patients with knee OA, affecting approximately one-third of patients worldwide [6,7]. Depression and pain share a bidirectional relationship: depressive symptoms can amplify the experience of pain, while poorly controlled pain increases the risk of depression [8,9]. Longitudinal studies in knee OA populations demonstrate that depression and anxiety not only co-occur with pain but also predict its persistence and worsening, underscoring their prognostic significance in disease progression [9].

Management of knee OA follows a biopsychosocial model, combining analgesic [10,11] and non-analgesic strategies, such as weight loss, physiotherapy, and cognitive behavioural therapies, to improve pain, function, and quality of life [10,11,12,13]. However, inadequate pain control has been linked to depression, reduced adherence to treatment, and impaired recovery [11,14,15,16]. Despite concerns regarding dependence and side effects, opioids are prescribed to up to 20% of patients within a year of diagnosis [17,18], with use particularly high among those awaiting surgery [19,20,21,22,23,24]. This reflects the broader interplay between biological pathology, psychological wellbeing, and social functioning, where depression may shape both the intensity of pain and the treatment strategies employed [15,16,18,25]

While the association between depression and pain in OA is increasingly recognised, its influence on analgesic prescribing and long-term outcomes remains insufficiently understood [26,27]. Addressing this gap is clinically relevant, as depressive symptoms may alter pain perception, engagement with treatment, and recovery trajectories, and optimising preoperative pain management in OA has been identified as a research priority by the James Lind Alliance partnership [26]. Therefore, this study aimed to evaluate the impact of baseline depressive symptoms on pain medication use, functional outcomes, and quality-of-life measures in patients with knee OA. We hypothesised that patients with depressive symptoms would demonstrate greater reliance on stronger pain medications, such as opioids, and experience poorer functional outcomes over two years.

## 2. Materials and Methods

### 2.1. Data Source

This retrospective cohort study used data from the Osteoarthritis Initiative (OAI) dataset, using the 4796 available patients between February 2004 and May 2006 [28].

Participants were included if they were aged 18 years or older, had no history of knee arthroplasty, and had available baseline Kellgren–Lawrence (KL) grade and SF-12 Mental Component Summary (MCS-12) data. Patients were excluded if they had missing baseline demographic information or underwent knee arthroplasty within the two-year study period. Records with missing values were excluded from the analysis.

We examined tabular variables for missing data, and records with missing values exceeding 60% were excluded under the assumption that the missing data occurred at random (n = 3680 observations, Table 1).

American Society of Anaesthesiologists (ASA) scores were calculated using guidelines outlined in the ASA Statement on Physical Status Classification [29]. Comorbidities were assigned one point each, as detailed in Supplementary Material Table S1. Smoking history was incorporated into the score, with non-smokers assigned a value of 0, and current or former smokers (quantified in pack-years) assigned a value of 1. Patients with a total score of 0 were classified as ASA 1, those with 2 as ASA 2, and those with 3+ as ASA 3+.

The KL grade is a validated scale for assessing OA severity, ranging from 0 to 4, used to measure the extent of OA [30], and values are provided as part of the OAI dataset [31]. The highest KL grade for either knee was recorded. This same principle was applied when considering the Knee injury and Osteoarthritis Outcome Score (KOOS) pain score, using the lowest, or most painful knee, as the final covariate. Body Mass Index (BMI), age and sex were also included as covariates, as detailed in Table 1.

### 2.2. Definitions and Outcomes

The primary outcomes were patterns of analgesia use over time and longitudinal changes in opioid use, comparing patients with and without depressive symptoms. Secondary outcomes included the proportion of patients achieving improvements exceeding the minimal clinically important difference (MCID) in the KOOS-QOL, the SF-12 Physical Component Summary (SF-12 PCS), and the Western Ontario and McMaster Universities Osteoarthritis Index (WOMAC), stratified by status. Knee OA was defined as tibiofemoral KL grade ≥ 1, or classified as at high risk of developing the disease, consistent with the OAI cohort population [31].

Depressive symptoms were defined using the SF-12 Mental Component Score (MCS-12), with a threshold of 45.6 or below, consistent with Vilagut et al.’s identification of the optimal cutoff for 30-day depressive disorders (sensitivity 86%, specificity 88%) [32]. The MCS-12 score reflects overall emotional wellbeing, incorporating domains such as vitality, social functioning, and emotional role limitation, and is often used as a proxy for depressive disorders. Lower scores indicate a greater burden of depressive symptoms. For the purposes of this study, patients were therefore categorised into two groups: (1) those with knee OA but without depressive symptoms (MCS-12 > 45.6), and (2) those with both knee OA and depressive symptoms (MCS-12 ≤ 45.6). This cutoff was selected because it aligns with the SF-12’s 4-week recall period.

Analgesic use was recorded at each OAI follow-up visit rather than as a single continuous exposure. For this study, medication use was assessed at baseline, Year 1, and Year 2. At each timepoint, participants were classified according to the analgesic category recorded in the OAI dataset, allowing temporal patterns to be evaluated rather than assuming ongoing use. Analgesics were stratified into the following categories: (1) NSAIDs and COX-2 inhibitors, (2) none, (3) topical salicylates, (4) opioids, and (5) combination of topical salicylates, NSAIDs, COX-2 inhibitors, and opioids.

With regard to the SF-12 PCS, we selected a MCID of 1.8 points, based on the anchor-based analysis by Clement et al., who evaluated over 2500 patients undergoing total knee arthroplasty for primary knee OA [33]. In this large, single-centre cohort, a change of 1.8 points identified as the threshold at which patients reported perceivable improvement in quality of life, independent of baseline case-mix factors.

For the WOMAC total score, we applied a 12-point threshold, derived from the anchor-based methodology reported by Clement et al. in their large TKA cohort study [34]. In this analysis, the MCID for WOMAC was established as 10 points, but increased to 12 points after multiple imputation to account for patients lost to follow-up. The 12-point threshold also corresponds to the minimum detectable change (MDC95), ensuring that observed improvements exceed measurement variability and reflect genuine, patient-perceived benefit.

We applied a 15-point threshold for the KOOS-QOL subscale, corresponding to the MCID identified by Roussel et al. in their prospective TKA cohort study of 218 patients [35]. This threshold provides a pragmatic benchmark for meaningful improvement, with TKA serving as the gold standard treatment in knee OA, though reliance on surgical cohorts may still limit direct generalizability to non-surgical populations.

### 2.3. Propensity Score Matching

**Figure 1:**
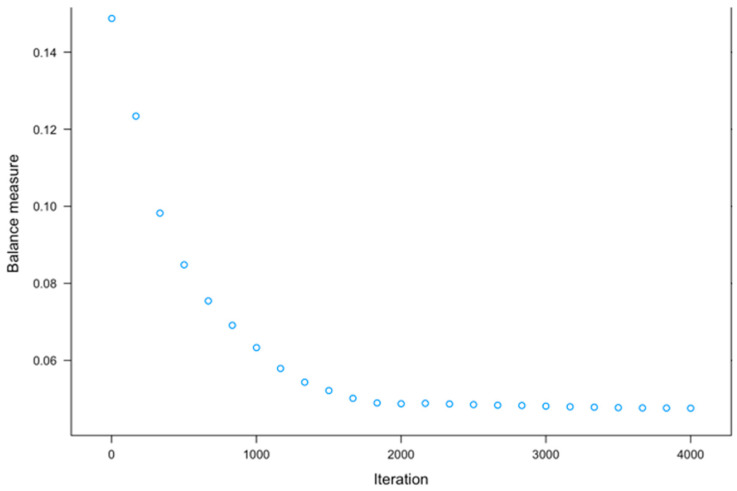
Pre-matching balance: Effect Size.

**Figure 2:**
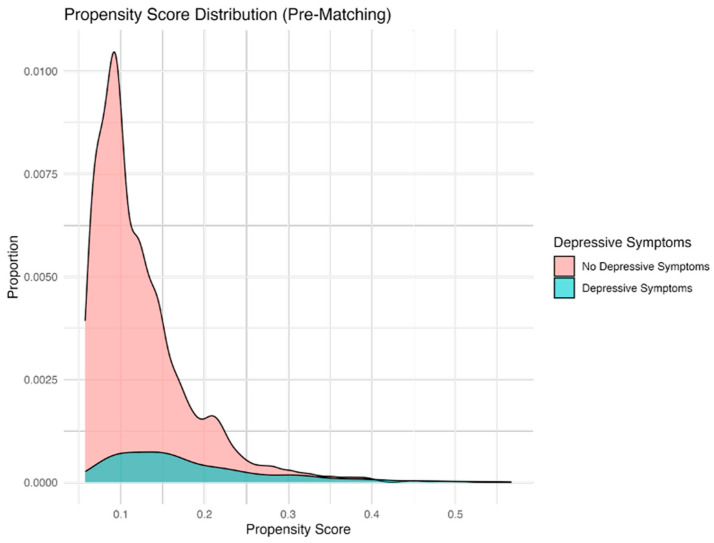
Pre-matching balance: Propensity.

**Figure 3:**
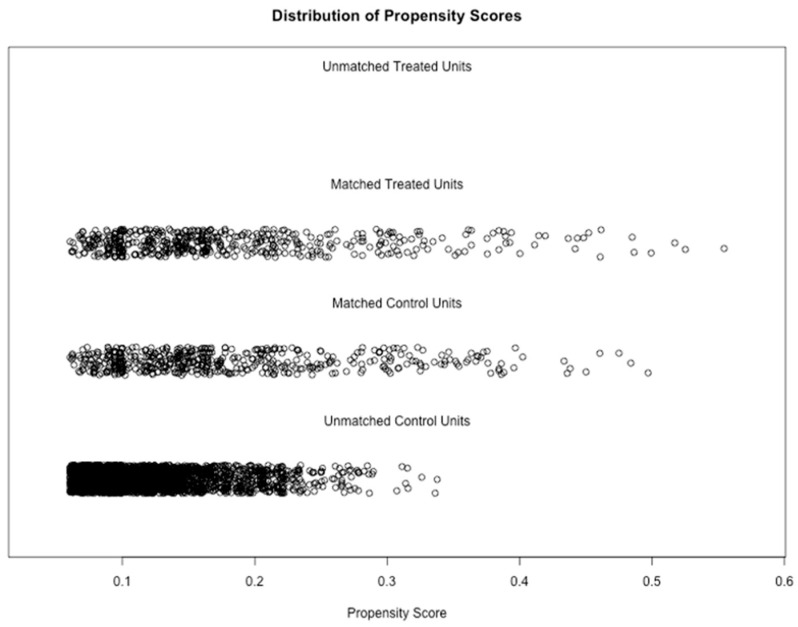
Distribution of propensity scores post-matching.

**Figure 4:**
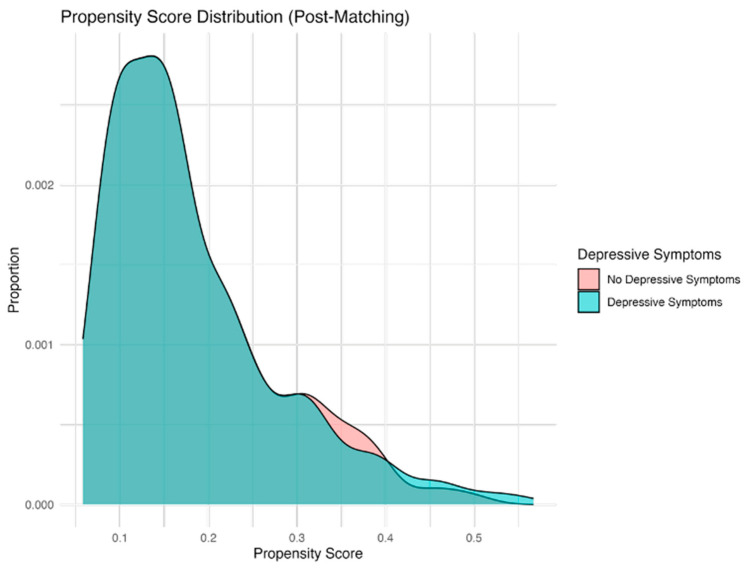
Distribution of propensity scores post-matching: Propensity.

Propensity score matching (PSM) is widely used in observational research as it reduces bias due to confounding by indication, allowing for comparisons between groups that more closely approximate those observed in randomised controlled trials [36]. The use of Gradient Boosting Machine (GBM), a non-parametric machine learning technique, provided a robust approach for estimating propensity scores in this study [37]. Through multiple iterations and varying interaction depths, the “twang” packages estimated propensity scores before matching, using a GBM framework. The “MatchIt” function was used to match the treatment group (patients with both knee OA and depressive symptoms) and control group (patients with only knee OA) based on propensity scores and calculated weights to balance both groups [37]. The optimal number of GBM iterations (3502) were used to minimise the discrepancies between covariates in both groups, optimising the effect size, also known as the absolute standardised mean difference, of the average treatment effect (Figure 1, Figure 2, Figure 3 and Figure 4). These estimates were used to compute a propensity score correlating to the probability of each observation belonging to the depressive symptom group. A one-to-one “optimal” matching approach using a “smallest” matching order algorithm was employed. This method prioritises minimising the total distance across all matched pairs with prior evidence suggests that optimal matching technique achieves covariate balance comparable to “nearest neighbour” matching, while potentially improving overall match quality [38]. “Smallest” matching order, ensures that those with the smallest propensity scores are matched first, thus maximising the similarity between the two groups [38]. The following covariates were included in the propensity score calculations: sex, age category, ASA grade, Kellgren–Lawrence grade, KOOS Pain score, BMI category, marital status, and ethnicity category, as seen in Table 1.

Figure 1 shows significant differences in covariates between depressed and non-depressed groups before matching, indicating the need for propensity score matching to ensure comparability and avoid biassed analyses. The optimal number of trees was identified at 3502 trees where the standardised mean difference was minimised (0.012) and further iterations showed stability.

Figure 2 displays distinct propensity score distributions for the groups with depressive symptoms (A) and those without depressive symptom (B), highlighting significant baseline imbalances and the necessity of matching for accurate treatment effect assessment.

Figure 3 displays distinct propensity score distributions for the groups with and without depressive symptoms, highlighting significant baseline imbalances and the necessity of matching for accurate treatment effect assessment.

Post-matching, Figure 4 shows overlapping propensity score distributions, confirming successful group balancing and enabling a fair comparison of outcomes between the groups with and without depressive symptoms.

#### 2.3.1. Diagnostic and Sensitivity Analyses for PSM

Both tabular and graphical diagnostics were employed to assess baseline balance between the cohorts with and without depressive symptoms, ensuring that the matching procedure produced comparable groups. The tabular review included crude and matched summary statistics with means, proportions, standardised mean differences (SMDs), and significance testing using χ^2^ tests for categorical covariates and *t*-tests for continuous covariates. Graphical evaluation was carried out with histograms, boxplots, and love plots to display the distribution of propensity scores before and after matching. After confirming satisfactory balance, unconditional univariable logistic regression models were used for binary outcomes, such as increase or decrease in opioid use. For continuous outcomes (KOOS Pain, WOMAC function, and SF-12 PCS), changes were dichotomized according to established minimally clinically important difference (MCID) thresholds, and logistic regression was used to estimate the odds of improvement or decline beyond these thresholds. All analyses were performed in R (version 4.4.0; R Foundation for Statistical Computing, Vienna, Austria) with the packages MatchIt (4.5.5), twang (2.6.1), optmatch (0.10.8), and cobalt (4.5.5).

#### 2.3.2. Crude Summary Statistics

A total of 3680 patients were included in the crude cohort (487 with depressive symptoms and 3193 without). There were statistically significant differences between the groups for all covariates, apart from KL grade (*p* = 0.471). Most covariates, including sex, age, ASA grade, KOOS pain, and marital status, differed at *p* < 0.001, while ethnicity (*p* = 0.013) and BMI (*p* = 0.005) also demonstrated significant between-group differences. Full baseline crude variables are presented in Table 1, and the distribution of propensity scores prior to matching is shown in Figure 1 and Figure 2.

#### 2.3.3. Matching

A total of 4000 Machine Learning based Gradient Boosting Machine (GBM) iterations were performed to minimise baseline covariate differences between the depression (treatment) and no-depression (control) groups. Optimal balance was achieved at 3502 trees, where the mean standardised effect size (ES) for the average treatment effect on the treated (ATT) was minimised (mean ES = 0.012, max ES = 0.031), thereby ensuring comparability between groups. The distribution of propensity scores before and after matching is illustrated in Figure 1 and Figure 2, and Supplementary Figures S1 and S2. A satisfactory graphical balance was achieved between the groups.

#### 2.3.4. Matched Summary Statistics

Using the propensity scores, it was possible to one-to-one match 487 patients with depressive symptoms to 487 patients without depressive symptoms. Baseline variables post matching are presented in Table 1. Overall, there were no statistically significant differences between groups (*p* > 0.05), with all standardised mean differences < 0.2. Between baseline and one year, 7.2% (n = 70) were lost to follow-up overall. This included 7.2% (n = 35) of patients with depressive symptoms and 7.2% (n = 35) of patients without depressive symptoms. By two years, the cumulative loss reached 11.2% (n = 109), comprising 11.3% (n = 55) of patients with depressive symptoms and 11.1% (n = 54) of patients without depressive symptoms. These matched cohorts were used to compare analgesic use, functional outcomes, and quality of life in this study.

### 2.4. Statistical Analysis

#### 2.4.1. Timepoint Analysis

The proportion of participants using each analgesic category was reported, stratified by depressive symptom status. To assess whether the proportion of participants using each analgesic changed significantly over time, we employed Generalised Estimating Equations (GEE) with a binomial distribution and a logit link function. GEE models account for the correlation of repeated measurements within individuals and were fitted using an exchangeable working correlation structure [39]. Individual GEE models were estimated for each analgesic class. Timepoint served as the primary predictor, and interaction terms with depressive symptoms included where appropriate to assess differential changes. Results are presented as the observed proportions of use at each timepoint, along with corresponding *p*-values derived from the GEE models to indicate statistical significance of changes over time. Statistical significance was defined as a two-sided *p*-value of less than 0.05.

#### 2.4.2. Logistic Regression

A series of multivariable logistic regression models were used to examine the association between depressive status and changes in opioid use and functional outcomes over time, adjusting for relevant covariates (Table 1). In the unmatched cohort, crude associations were explored using univariable logistic regression, while in the matched cohort, multivariable logistic regression was applied, adjusting for the covariates used in matching to account for any residual confounding. All models were specified as binary logistic regression with a logit link function. Opioid use was categorised as either opioid or opioid combination use versus non-opioid analgesics, such as NSAIDs, COX-2 inhibitors, or topical salicylates, and changes over time were evaluated accordingly. Logistic regression was applied to estimate the odds of achieving MCID-level improvements, with linear regression used for continuous score changes.

Odds ratios (ORs) with 95% confidence intervals (CIs) were reported for all models using the profile likelihood method, with the *p*-values derived from Wald tests. Statistical significance was defined as a two-sided *p*-value < 0.05. ORs greater than 1 indicated outcomes more likely among individuals with depressive symptoms.

### 2.5. Outcome Analysis

Continuous secondary outcomes were assessed using paired *t*-tests for normally distributed data and Wilcoxon signed-rank tests otherwise, with normality evaluated via visual inspection of box plots and the Shapiro–Wilk test.

## 3. Results

### 3.1. Analgesia Usage

#### 3.1.1. Temporal Trends

In the crude (unmatched) cohort, individuals with depressive symptoms demonstrated a modest increase in the proportion reporting no analgesic use, rising from 73.3% to 78.6% (*p* = 0.027), with no statistically significant changes in the use of NSAIDs/COX-2 inhibitors, opioids, topical salicylates, or combination therapies. Among those without depressive symptoms, the proportion reporting no analgesic use increased significantly from 79.1% at baseline to 85.0% at Year 2 (*p* < 0.001), accompanied by a decline in NSAID/COX-2 inhibitor use (14.0% to 9.6%; *p* < 0.001) and topical salicylates (2.6% to 1.0%; *p* = 0.002), while other categories remained stable (Table 2).

In contrast, the matched cohort revealed more attenuated temporal patterns. Among individuals with depressive symptoms, the proportion reporting no analgesic use increased significantly from 73.3% at baseline to 78.6% at Year 2 (*p* = 0.027). However, use of NSAIDs/COX-2 inhibitors, opioids, topical salicylates, and combination therapies remained statistically unchanged over the two-year period. Among matched individuals without depressive symptoms, a transient increase in the proportion reporting no analgesic use was observed at Year 1 (76.8% to 79.7%; *p* = 0.041), though this was not sustained through Year 2 (81.5%; *p* = 0.057), with no significant changes across other analgesic categories (Table 3).

#### 3.1.2. Logistic Regression

In both crude and propensity-matched analyses, living with depressive symptoms was not significantly associated with increased opioid use over the two-year period. Crude analysis showed no significant differences (Year 1 OR 1.156; 95% CI 0.608–2.028; *p* = 0.634, Year 2 OR 1.291; 95% CI 0.736–2.135; *p* = 0.343), suggesting no clear relationship between living with depressive symptoms and changes in opioid use over two years. In the matched cohort, the odds of initiating opioid use appeared lower for participants living with depressive symptoms at both Year 1 (OR 0.706; 95% CI 0.334–1.451; *p* = 0.349) and Year 2 (OR 0.887; 95% CI 0.450–1.731; *p* = 0.725), but without statistical significance (Table 4).

### 3.2. Secondary Outcomes

#### 3.2.1. Temporal Trends

In the crude cohorts, patients with depressive symptoms reported significantly worse symptoms in all domains including pain, function (WOMAC), physical health (SF-12 PCS), and quality of life (KOOS QoL) scores across all three timepoints (*p* < 0.001). However, in the matched cohort, these outcomes were similar between groups, with no statistically significant differences across timepoints (*p* > 0.05) (Supplementary Tables S2–S4).

#### 3.2.2. Logistic Regression

Crude analysis showed that depressive symptoms were associated with broadly less favourable outcomes (Table 4). These individuals had significantly higher odds of KOOS QOL decline ≥ MCID at Year 2 (OR 1.438; 95% CI 1.055–1.931; *p* = 0.018), lower odds of SF-12 PCS improvement ≥ MCID at Year 1 (OR 0.784; 95% CI 0.629–0.973; *p* = 0.029), and greater odds of SF-12 PCS decline ≥ MCID at both Year 1 (OR 2.066; 95% CI 1.693–2.523; *p* < 0.001) and Year 2 (OR 2.144; 95% CI 1.750–2.631; *p* < 0.001). The isolated finding of higher odds of WOMAC functional improvement ≥ MCID at year 1 was not sustained at year 2 (*p* > 0.05).

In contrast, propensity score-matched analysis revealed a differentiated pattern. Depressive symptoms were associated with significantly lower odds of SF-12 PCS improvement ≥ MCID at Year 1 (OR 0.696; 95% CI 0.523–0.926; *p* = 0.013), and a strong association with greater odds of PCS-12decline ≥ MCID at both Year 1 (OR 2.013; 95% CI 1.539–2.640; *p* < 0.001) and Year 2 (OR 1.930; 95% CI 1.469–2.540; *p* < 0.001). Conversely, these patients had markedly increased odds of WOMAC functional improvement ≥MCID at Year 1 (OR 2.674; 95% CI 1.745–4.177; *p* < 0.001), and numerically higher (but non-significant) odds of improvement ≥ MCID at Year 2 (OR 1.464; 95% CI 0.962–2.244; *p* = 0.077), and reduced odds of WOMAC decline ≥ MCID at both Year 1 (OR 0.686; 95% CI 0.495–0.948; *p* = 0.023) and Year 2 (OR 0.721; 95% CI 0.527–0.983; *p* = 0.039).

## 4. Discussion

In this propensity score-matched analysis of OAI data, we found that patients with depressive symptoms did not experience worse clinical outcomes or increased opioid use compare with those without such symptoms. Although patients with depressive symptoms reported lower physical health scores at baseline, they demonstrated less decline in physical functional and even some functional improvement over time. Importantly, depressive symptoms were not associated with worse clinical outcomes or increased opioid use, which may reassure clinicians concerned about escalation in this group. These patterns highlight the complex interplay between pain, function, and mood, and indicate that depression status alone may not be a sufficient proxy for predicting opioid use or recovery trajectories in patients with knee OA.

Optimising preoperative analgesia while addressing mental health and pain levels is crucial for improving surgical outcomes and long-term recovery [40]. This is especially relevant in the context of prolonged surgical waiting lists, as highlighted in a recent report on NHS backlogs, where patients may require sustained and adaptive pain management strategies [21]. At a population-level, Taqi et al. analysed over 8.9 million prescriptions for 117,637 patients with knee OA from the UK Clinical Practice Research Datalink (CPRD) and reported rising overall analgesic prescribing between 2000 and 2014, with a marked 57.7% decline in NSAIDs [41]. Similar trends were observed in other European countries [41,42]. By contrast, our matched cohort did not demonstrate declining NSAID/COX-2 inhibitor use but did show rising rates of no-analgesia rates among patients with depressive symptoms. Importantly, previous studies did not explore prescribing patterns according to depression status, which may have resulted in the oversight of systematic differences in these groups [41,42]. Prior work has shown that individuals with coexisting depression and pain report greater symptom burden and altered treatment patterns, including differences in analgesic prescribing [43,44]. The increase in no-analgesia use in patients with depressive symptoms may reflect alternative pain-coping strategies, altered prescribing behaviours, or greater reliance on non-pharmacological approaches, rather than mirroring the population-level reduction in NSAID prescribing. Given the global rise in both OA and depression prevalence [45,46], this distinction has important clinical implications. Patients with depressive symptoms may sustain functional improvement without escalating pharmacological use, whereas patients without depressive symptoms may decline despite stable medication use. This highlights the need for tailored, proactive pain management, and future works should assess medication patterns with functional outcomes in patients with depressive symptoms. A multidisciplinary team (MDT) approach that integrates pain specialists and mental health professionals, may aid in providing comprehensive preoperative care [47]. Among patients with depressive symptoms, greater use of psychological and behavioural interventions may reduce reliance on analgesics and explain the observed rise in no-analgesia, though this was not directly captured in the OAI dataset [27]. Pain specialists can optimise regimens while avoiding overprescription, and mental health professionals can provide cognitive behavioural therapy (CBT), counselling, or pharmacological interventions to improve psychological readiness for surgery. In the context of prolonged surgical waits, MDT-led prehabilitation clinics may sustain function, manage distress, and limit inappropriate opioid escalation, ultimately improving outcomes, and quality of life for patients with knee OA.

Opioid use in knee OA remains a significant concern, particularly among patients with comorbid depression. In Sweden, one in four knee OA patients receive an opioid prescription annually, with OA-related comorbidities contributing to 12% of new dispensations [48]. This is particularly concerning given the well-documented risks of opioid dependency and the heightened pain sensitivity often observed in depressed patients, which could potentially drive increased opioid reliance [49]. This vulnerability is thought to arise from shared neurobiological pathways, including serotonergic and noradrenergic dysregulation, while persistent pain may in turn exacerbate depressive states through functional decline and social withdrawal [50,51]. Despite these concerns, our study did not demonstrate higher opioid use among patients with depressive symptoms, suggesting that depression alone does not predispose to greater opioid dependency. This may reflect differences in study populations, prescribing behaviours, or greater engagement with non-pharmacological strategies among this group. Of note, our results contrast with Vina et al., who reported that moderate-to-severe depression was associated with significantly higher odds of opioid initiation in a US-based cross-sectional analysis of 360 primary care patients with chronic knee OA [27]. Importantly, their findings did not show a significant preference for opioids over non-opioid analgesics once treatment had begun. Together with our findings, which examined a broader spectrum of depressive symptoms using Vilagut et al.’s MCS-12 threshold [32], this suggests that opioid dependence may depend on depression severity: severe depressive may drive initiation, whereas milder symptoms, as shown in our study, do not appear to increase dependency over time. These insights reinforce the need for a biopsychosocial model of care that addresses psychological vulnerability and social context alongside physical pain, to minimise unnecessary opioid initiation and support sustainable pain management.

Regarding functional outcomes, a meta-analysis by Osani et al. of 18 RCTs involving over 9000 patients found that opioids only provided marginal and transient benefits in knee OA, with small effect sizes for pain relief and function, peaking at four weeks, with no sustained impact on quality of life [52]. Our study aligns with this evidence, as opioid use remained stable over two years in both cohorts, supporting the limited efficacy of opioids in OA management, but more evidence is required to determine whether stability in use directly reflects lack of clinical benefit [53]. Although we observed higher odds of WOMAC functional improvement at year 1 among patients with depressive symptoms, this effect was not sustained at year 2, likely reflecting regression to the mean, ceiling effects, or unmeasured confounders such as comorbidity and physical activity. Notably, patients with depressive symptoms who entered the study with worse baseline pain and function, were more likely to achieve clinically meaningful WOMAC gains, while those without depressive symptoms showed less scope for improvement and even declined in SF-12 PCS. The lower odds of SF-12 PCS improvement among patients with depressive symptoms likely reflect differences in instrument focus and responsiveness. Although the SF-12 PCS is validated for use in knee OA, its brevity as a short-form tool may reduce sensitivity to change. In addition, it is a generic, norm-based composite that captures broader domains such as physical functioning, role limitations, bodily pain, and general health [54]. In contrast, the WOMAC is disease-specific and more sensitive to knee-related changes [55]. As a result, knee-specific gains may be more readily detected on the WOMAC, whereas improvements can appear diluted on the SF-12 PCS by comorbidities, wider health perceptions, and mood-related influences on pain and role-limitation items [56]. These patterns may also reflect greater engagement with supportive interventions, or mood-related improvements that enhance pain perception and functional reporting, even without structural changes. The absence of parallel gains in KOOS-QOL underscores that functional improvements do not necessarily translate into better perceived quality of life. Overall, these findings emphasises that functional outcomes in knee OA must be interpreted within a biopsychosocial framework, accounting for baseline severity, mood, and the interdependence of pain and function. If improvements among patients with depressive symptoms partly reflect engagement with mood-supportive interventions, this possibility warrants further study to determine whether similar strategies might benefit patients with knee OA that do not experience depressive symptoms through a similar mechanism.

The main strength of this study lies in the use of propensity score matching, which minimised selection bias and controlled for confounding, ensuring that patients with knee OA and depressive symptoms were comparable to non-depressed counterparts. However, there are several limitations to report. First, the matched cohort included only 886 patients, which may limit statistical power and generalisability. The study was based on a US cohort, and prescribing patterns, access to physiotherapy, and mental health services may differ across healthcare systems. As such, the findings may not fully generalise to other regions with different clinical practices, insurance structures, or cultural approaches to pain management and depression. Methodological constraints of the OAI dataset also restricted analyses: ASA grade was estimated from available variables (Supplementary Table S1) rather than directly assessed, KL grades were collapsed to the highest value per patient, and missing values were excluded from follow-up analyses [28,29].

In addition, it should be noted that The OAI cohort includes individuals with KL grades 0–4, so our analysis encompassed both established OA and those only at risk. However, this reflects the OAI design and allows evaluation across the full spectrum of disease progression. This mixed population may limit the direct applicability of our findings to patients with clinically confirmed, symptomatic OA. The lack of detailed information on antidepressant type or more granular analgesic usage restricted subgroup exploration. Regression to the mean may also have influenced this outcome. Finally, MCIDs and cut-offs for functional outcomes were derived primarily from papers analysing TKA cohorts, which may reduce generalisability to non-surgical populations [57,58].

## 5. Conclusions

This study highlights distinct trajectories in analgesic use and outcomes between patients with and without depressive symptoms, with stable opioid use across groups over two years, but rising use of no-analgesia and functional gains among those with depressive symptoms, contrasted with physical decline in non-depressed patients. These findings extend our understanding of the osteoarthritis–depression pain syndrome and underscore the need for adopting a biopsychosocial, patient-centred model of care. Clinically, this means combining tailored pharmacological regimens with mental health support and non-opioid multimodal interventions to optimise symptom control, improve adherence, reduce pain intensity, and enhance recovery. A novel aspect of this work is the application of machine learning-based propensity score matching to generate well-balanced comparison groups, allowing clearer interpretation of the independent effect of depressive symptoms on outcomes. By focusing on depressive symptoms rather than only diagnosed mood disorders, this study captures a broader and clinically relevant population and demonstrates that depressive symptoms alone may not lead to opioid escalation. In the context of long surgical waiting lists and increasing pressure on health systems, optimising preoperative pain management aligns with the James Lind Alliance’s priorities and remains both a clinical and policy imperative, with the need to combine tailored pharmacological regimens, mental health support, and non-opioid multimodal interventions in patients with OA.

## Figures and Tables

**Table 1:** Demographics Table.

	Crude Cohort				Matched Cohort			
	Depressive Symptoms (N = 487)	No Depressive Symptoms (N = 3193)	*p*	SMD	Depressive Symptoms (N = 487)	No Depressive Symptoms (N = 487)	*p*	SMD
**Sex**			**<0.001**	0.67			0.735	0.03
Male	167 (34.3%)	1366 (42.8%)			161 (33.1%)	167 (34.3%)		
Female	320 (65.7%)	1827 (57.2%)			326 (66.9%)	320 (65.7%)		
**Age**			**<0.001**	3.07			0.716	0.03
45–55	198 (40.7%)	940 (29.4%)			197 (40.5%)	198 (40.7%)		
55–65	179 (36.8%)	1025 (32.1%)			170 (34.9%)	179 (36.8%)		
65–80	110 (22.6%)	1228 (38.5%)			120 (24.6%)	110 (22.6%)		
**ASA**			**<0.001**	3.28			0.475	0.01
1	121 (24.8%)	1044 (32.7%)			132 (27.1%)	121 (24.8%)		
2	266 (54.6%)	1771 (55.5%)			247 (50.7%)	266 (54.6%)		
3+	100 (20.5%)	378 (11.8%)			108 (22.2%)	100 (20.5%)		
**KL Grade**			0.471	2.36			0.557	0.10
0	202 (41.5%)	1304 (40.8%)			130 (26.7%)	148 (30.4%)		
1	92 (18.9%)	568 (17.8%)			73 (15.0%)	74 (15.2%)		
2	127 (26.1%)	799 (25.0%)			152 (31.2%)	154 (31.6%)		
3	58 (11.9%)	432 (13.5%)			103 (21.1%)	87 (17.9%)		
4	8 (1.6%)	90 (2.8%)			29 (6.0%)	24 (4.9%)		
**KOOS Pain**			**<0.001**	6.14			0.068	0.11
Mean (SD)	72.7 (21.3)	81.4 (17.8)			70.4 (21.4)	72.7 (21.3)		
Median (IQR)	77.8 (58.3–88.9)	86.1 (72.2–97.2)			75.0 (55.6–86.1)	77.8 (56.2–88.9)		
**BMI**			**0.005**	4.97			0.854	0.01
Underweight (<18.5 kg/m^2^)	2 (0.4%)	7 (0.2%)			3 (0.6%)	2 (0.4%)		
Normal (18.5–24.9 kg/m^2^)	114 (23.4%)	799 (25.0%)			109 (22.4%)	114 (23.4%)		
Overweight (25.0–29.9 kg/m^2^)	169 (34.7%)	1312 (41.1%)			180 (37.0%)	169 (34.7%)		
Obese (≥30 kg/m^2^)	202 (41.5%)	1075 (33.7%)			195 (40.0%)	202 (41.5%)		
**Ethnicity**			**0.013**	2.27			0.494	0.06
White/Caucasian	369 (75.8%)	2623 (82.1%)			352 (72.3%)	369 (75.8%)		
Black/African	106 (21.8%)	500 (15.7%)			124 (25.5%)	106 (21.8%)		
Hispanic/Latino	2 (0.4%)	14 (0.4%)			1 (0.2%)	2 (0.4%)		
Asian	3 (0.6%)	25 (0.8%)			1 (0.2%)	3 (0.6%)		
Other	7 (1.4%)	31 (1.0%)			9 (1.8%)	7 (1.4%)		
**Marital Status**			**<0.001**	4.41			0.959	0.02
Never Married	67 (13.8%)	264 (8.3%)			64 (13.1%)	67 (13.8%)		
Currently Married	273 (56.1%)	2247 (70.4%)			274 (56.3%)	273 (56.1%)		
Other	147 (30.2%)	682 (21.4%)			149 (30.6%)	147 (30.2%)		

Footnote: Variables included sex, age, American Society of Anesthesiologists (ASA) physical status classification, Kellgren–Lawrence (KL) grade, Knee injury and Osteoarthritis Outcome Score (KOOS) pain subscale, body mass index (BMI), ethnicity, and marital status. Sex was defined as male or female. Age was grouped into 45–55, 55–65, and 65–80 years. ASA grade was determined according to ASA guidelines, with each comorbidity (heart failure, myocardial infarction, stroke, asthma, chronic lung disease, diabetes, impaired kidney function) contributing one point, and smoking history scored as 0 for non-smokers and 1 for current or former smokers; patients with a total score of 0 were classified as ASA 1, those with a score of 2 as ASA 2, and those with a score of 3 or greater as ASA 3+, while BMI was categorised separately but not included in ASA scoring. KL grades ranged from 1 (doubtful narrowing of joint space and possible osteophytic lipping) to 4 s (severe joint space narrowing, large osteophytes, marked sclerosis, and definite bony deformity). KOOS pain was assessed using the validated pain subscale of the Knee injury and Osteoarthritis Outcome Score. BMI was categorised as underweight (<18.5 kg/m^2^), normal (18.5–24.9), overweight (25.0–29.9), and obese (≥30, with subcategories Obese I: 30–34.9, Obese II: 35–39.9, and Obese III: 40–59.9). Ethnicity was self-reported as White/Caucasian, Black/African, Hispanic/Latino, Asian, or other. Marital status was classified as never married, currently married, or other (including divorced, widowed, separated, or partnered without legal marriage). All significant *p*-values are shown in bold.

**Table 2:** Granular Longitudinal Analysis of Analgesia Usage in the Crude Cohorts, Stratified by Depression Status.

		Cohort with Depression				Cohort Without Depression			
Analgesic	Comparison	Baseline %	Year 1%	Year 2%	Significance	Baseline %	Year 1%	Year 2%	Significance
**NSAIDs and COX2**	*Baseline* vs. *Year 1*	15.2	12.5	12.5	0.230	14	10.7	9.6	**<0.001**
	*Baseline* vs. *Year 2*	15.2	12.5	12.5	0.327	14	10.7	9.6	**<0.001**
	*Year 1* vs. *Year 2*	15.2	12.5	12.5	>0.999	14	10.7	9.6	0.086
**None**	*Baseline* vs. *Year 1*	73.3	77.6	78.6	**0.040**	79.1	83.4	85	**<0.001**
	*Baseline* vs. *Year 2*	73.3	77.6	78.6	**0.027**	79.1	83.4	85	**<0.001**
	*Year 1* vs. *Year 2*	73.3	77.6	78.6	0.850	79.1	83.4	85	**0.027**
**Topical** **Salicylates**	*Baseline* vs. *Year 1*	2.1	2.1	1	>0.999	2.6	1.6	1	**0.002**
	*Baseline* vs. *Year 2*	2.1	2.1	1	0.230	2.6	1.6	1	**<0.001**
	*Year 1* vs. *Year 2*	2.1	2.1	1	0.151	2.6	1.6	1	0.091
**Opioids**	*Baseline* vs. *Year 1*	5.1	5.1	5.1	>0.999	2	2.3	2.5	0.497
	*Baseline* vs. *Year 2*	5.1	5.1	5.1	>0.999	2	2.3	2.5	0.198
	*Year 1* vs. *Year 2*	5.1	5.1	5.1	>0.999	2	2.3	2.5	0.770
**Combination of Topical Salicylates, NSAIDs, COX2 and Opioids**	*Baseline* vs. *Year 1*	4.3	2.7	2.7	0.208	2.3	2	1.8	0.503
	*Baseline* vs. *Year 2*	4.3	2.7	2.7	0.237	2.3	2	1.8	0.137
	*Year 1* vs. *Year 2*	4.3	2.7	2.7	>0.999	2.3	2	1.8	0.623

**Footnote: Due to small sample sizes, “other/unspecified” analgesia was not analysed.** All significant *p*-values are shown in bold.

**Table 3:** Granular Longitudinal Analysis of Analgesia Usage in the Matched Cohorts, Stratified by Depression Status.

		Cohort with Depression				Cohort Without Depression			
Analgesic	Comparison	Baseline %	Year 1%	Year 2%	Significance	Baseline %	Year 1%	Year 2%	Significance
**NSAIDs and COX2**	*Baseline* vs. *Year 1*	15.2	12.5	12.5	0.2296	14.4	12.3	10.9	0.4122
	*Baseline* vs. *Year 2*	15.2	12.5	12.5	0.3273	14.4	12.3	10.9	0.0949
	*Year 1* vs. *Year 2*	15.2	12.5	12.5	>0.9999	14.4	12.3	10.9	0.5493
**None**	*Baseline* vs. *Year 1*	73.3	77.6	78.6	**0.0399**	76.8	79.7	81.5	0.2774
	*Baseline* vs. *Year 2*	73.3	77.6	78.6	**0.0267**	76.8	79.7	81.5	0.0571
	*Year 1* vs. *Year 2*	73.3	77.6	78.6	0.8504	76.8	79.7	81.5	0.5241
**Topical** **Salicylates**	*Baseline* vs. *Year 1*	2.1	2.1	1	>0.9999	2.7	1.6	1.4	0.4521
	*Baseline* vs. *Year 2*	2.1	2.1	1	0.2302	2.7	1.6	1.4	0.3831
	*Year 1* vs. *Year 2*	2.1	2.1	1	0.1514	2.7	1.6	1.4	0.9585
**Opioids**	*Baseline* vs. *Year 1*	5.1	5.1	5.1	>0.9999	3.5	3.9	3.9	0.9123
	*Baseline* vs. *Year 2*	5.1	5.1	5.1	>0.9999	3.5	3.9	3.9	0.9188
	*Year 1* vs. *Year 2*	5.1	5.1	5.1	>0.9999	3.5	3.9	3.9	>0.9999
**Combination of Topical** **Salicylates, NSAIDs, COX2 and Opioids**	*Baseline* vs. *Year 1*	4.3	2.7	2.7	0.2081	2.7	2.5	2.3	0.9714
	*Baseline* vs. *Year 2*	4.3	2.7	2.7	0.2371	2.7	2.5	2.3	0.8716
	*Year 1* vs. *Year 2*	4.3	2.7	2.7	>0.9999	2.7	2.5	2.3	0.9511

Footnote: Due to small sample sizes, “other/unspecified” analgesia was not analysed. All significant *p*-values are shown in bold.

**Table 4:** Odds Ratios of Outcomes.

Outcomes	Propensity Matched Comparisons		Crude Comparisons	
	Odds Ratio (95% CI)	Significance	Odds Ratio (95% CI)	Significance
**KOOS QOL Improvement Year 1**	0.788 (0.575–1.077)	0.136	1.069 (0.834–1.359)	0.592
**KOOS QOL Improvement Year 2**	0.764 (0.559–1.043)	0.091	1.060 (0.828–1.347)	0.636
**KOOS QOL Decline Year 1**	1.225 (0.801–1.883)	0.350	1.274 (0.925–1.725)	0.126
**KOOS QOL Decline Year 2**	1.522 (0.993–2.353)	0.056	1.438 (1.055–1.931)	**0.018**
**SF-12 Physical Improvement Year 1**	0.696 (0.523–0.926)	**0.013**	0.784 (0.629–0.973)	**0.029**
**SF-12 Physical Improvement Year 2**	0.746 (0.555–1.001)	0.051	0.837 (0.667–1.046)	0.122
**SF-12 Physical Decline Year 1**	2.013 (1.539–2.640)	**<0.001**	2.066 (1.693–2.523)	**<0.001**
**SF-12 Physical Decline Year 2**	1.930 (1.469–2.540)	**<0.001**	2.144 (1.750–2.631)	**<0.001**
**WOMAC Improvement Year 1**	2.674 (1.745–4.177)	**<0.001**	2.143 (1.621–2.805)	**<0.001**
**WOMAC Improvement Year 2**	1.464 (0.962–2.244)	0.077	1.498 (1.103–2.006)	**0.008**
**WOMAC Decline Year 1**	0.686 (0.495–0.948)	**0.023**	1.183 (0.910–1.521)	0.200
**WOMAC Decline Year 2**	0.721 (0.527–0.983)	**0.039**	1.410 (1.098–1.796)	**0.006**
**Increased Opioid Use Year 1**	0.706 (0.334–1.451)	0.349	1.156 (0.608–2.028)	0.634
**Increased Opioid Use Year 2**	0.887 (0.450–1.731)	0.725	1.291 (0.736–2.135)	0.343

Footnote: All outcomes are gives as changes of a greater magnitude than the MCIDs. Odds Ratios > 1 favour patients with depressive symptoms. All significant *p*-values are shown in bold.

## Data Availability

The datasets presented in this article are not readily available because they are sourced from the controlled access datasets distributed from the Osteoarthritis Initiative (OAI), a data repository housed within the NIMH Data Archive (NDA). OAI is a collaborative informatics system created by the National Institute of Mental Health and the National Institute of Arthritis, Musculoskeletal and Skin Diseases (NIAMS) to provide a worldwide resource to quicken the pace of biomarker identification, scientific investigation and OA drug development. Dataset identifier: https://nda.nih.gov/oai.

## Supplementary Materials

Supporting information can be downloaded at: https://www.mdpi.com/article/10.3390/bioengineering13010063/s1, Supplementary Figure S1: Standardised Mean Differences Before and After Matching; Supplementary Figure S2: Balance Plot of the Crude and Matched Data; Table S1: Factors used in ASA definition; Table S2: Baseline Outcomes; Table S3: Outcomes at 1 Year; Table S4: Outcomes at 2 Years.

## Abbreviations

ASA	American Society of Anesthesiologists
ATT	Average Treatment Effect on the Treated
BMI	Body Mass Index
CBT	Cognitive Behavioural Therapy
CI	Confidence Interval
COX-2	Cyclooxygenase-2
CPRD	Clinical Practice Research Datalink
GBM	Gradient Boosting Machine
GEE	Generalised Estimating Equations
KL	Kellgren–Lawrence
KOOS	Knee Injury and Osteoarthritis Outcome Score
KOOS-QOL	Knee Injury and Osteoarthritis Outcome Score—Quality of Life
LOS	Length of Stay
MCID	Minimal Clinically Important Difference
MCS-12	Mental Component Summary (12-Item Short Form)
MDT	Multidisciplinary Team
MDC95	Minimum Detectable Change at 95% Confidence
NSAID	Non-steroidal Anti-inflammatory Drug
NHS	National Health Service
OAI	Osteoarthritis Initiative
OR	Odds Ratio
PCS-12	Physical Component Summary (12-Item Short Form)
PSM	Propensity Score Matching
RCT	Randomised Controlled Trial
SF-12	Short Form-12 Health Survey
SMD	Standardised Mean Difference
TKA	Total Knee Arthroplasty
WOMAC	Western Ontario and McMaster Universities Osteoarthritis Index
