# Supplementary material for: The Link Between Depression, Analgesia Usage and Function in Osteoarthritis: A Propensity Score-Matched Analysis from the Osteoarthritis Initiative Cohort
Source: Bioengineering (Basel). 2026 Jan 6;13(1):63. doi: 10.3390/bioengineering13010063 (PMC12838159; doi:10.3390/bioengineering13010063)
Supplement: Supplementary file 1 [file bioengineering-13-00063-s001.zip › bioengineering-4016167-supplementary.pdf]

Supplementary Tables

**Table s1: Factors used in ASA definition**
Comorbidities
Heart failure<br>Heart attack<br>Stroke<br>Asthma<br>Lung disease<br>Diabetes<br>Kidney function
Smoking
No smoking history = 0<br>0 < Pack years ≤ 5 = 1<br>5 < Pack years ≤ 19 = 1<br>Pack years ≥ 20 = 1
BMI
Underweight = 0 ≤ BMI < 18.5<br>Normal = 18.5 ≤ BMI < 25<br>Overweight = 25 ≤ BMI < 30<br>Obese 1 = 30 ≤ BMI < 35<br>Obese 2 = 35 ≤ BMI < 40<br>Obese 3 = 40 ≤ BMI < 60

**Table s2: Baseline Outcomes**						
	**Matched Cohort**			**Crude Cohort**		
**Variable**	**Depressed (N=486)**	**Not Depressed (N=485)**	**Significance**	**Depressed (N=486)**	**Not Depressed (N=3187)**	**Significance**
Analgesic Use			0.426			**<0.001**
None	357 (73.5%)	372 (76.7%)		357 (73.5%)	2522 (79.1%)	
Topical Salicylates	10 (2.1%)	13 (2.7%)		10 (2.1%)	84 (2.6%)	
NSAIDs & COX2	74 (15.2%)	70 (14.4%)		74 (15.2%)	446 (14.0%)	
Opioids	24 (4.9%)	17 (3.5%)		24 (4.9%)	63 (2.0%)	
Combination	21 (4.3%)	13 (2.7%)		21 (4.3%)	72 (2.3%)	
Other/Unspecified	0 (0%)	0 (0%)		0 (0%)	0 (0%)	
WOMAC			0.135			**<0.001**
Median (IQR)	15.03 (6–33.28)	18.62 (6.19–35)		15.0 (6.0–33.3)	8.0 (2.0–21.0)	
SF-12 Physical			<0.001			0.997
Median (IQR)	51.03 (42.45–57.53)	49.64 (39.97–55.02)		51.0 (42.4–57.5)	52.5 (45.8–56.1)	
KOOS QOL			0.943			**<0.001**
Median (IQR)	62.5 (43.75–75)	62.5 (43.75–75)		62.5 (43.8–75.0)	68.8 (56.2–87.5)	

Table s3: Outcomes at 1 Year						
	**Matched Cohort**			**Crude Cohort**		
**Variable**	**Depressed (N=451)**	**Not Depressed (N=451)**	**Significance**	**Depressed (N=451)**	**Not Depressed (N=3043)**	**Significance**
Analgesic Use			0.783			<0.001
None	350 (77.6%)	354 (78.5%)		350 (77.6%)	2525 (83.0%)	
Topical Salicylates	10 (2.2%)	8 (1.8%)		10 (2.2%)	49 (1.6%)	
NSAIDs & COX2	53 (11.8%)	59 (13.1%)		53 (11.8%)	337 (11.1%)	
Opioids	25 (5.5%)	18 (4.0%)		25 (5.5%)	71 (2.3%)	
Combination	13 (2.9%)	12 (2.7%)		13 (2.9%)	61 (2.0%)	
Other/Unspecified	0 (0%)	0 (0%)		0 (0%)	0 (0%)	
WOMAC			0.185			**<0.001**
Median (IQR)	14 (3–31)	12 (2.2–28)		14.0 (3.0–31.0)	6.0 (1.0–17.0)	
SF-12 Physical			0.985			**<0.001**
Median (IQR)	48.24 (40.26–55.51)	50.34 (39.41–55.86)		48.2 (40.3–55.5)	52.5 (45.0–56.1)	
KOOS QOL			0.091			**<0.001**
Median (IQR)	68.75 (50–81.25)	68.75 (50–87.5)		68.8 (50.0–81.2)	75.0 (62.5–93.8)	

**Table s4: Outcomes at 2 Years**						
	**Matched Cohort**			**Crude Cohort**		
**Variable**	**Depressed (N=431)**	**Not Depressed (N=432)**	**Significance**	**Depressed (N=431)**	**Not Depressed (N=2920)**	**Significance**
Analgesic Use			0.931			0.002
None	334 (77.5%)	344 (79.6%)		334 (77.5%)	2466 (84.5%)	
Topical Salicylates	5 (1.2%)	6 (1.4%)		5 (1.2%)	28 (1.0%)	
NSAIDs & COX2	57 (13.2%)	52 (12.0%)		57 (13.2%)	297 (10.2%)	
Opioids	23 (5.3%)	19 (4.4%)		23 (5.3%)	78 (2.7%)	
Combination	12 (2.8%)	11 (2.5%)		12 (2.8%)	51 (1.7%)	
Other/Unspecified	0 (0%)	0 (0%)		0 (0%)	0 (0%)	
WOMAC			0.348			<0.001
Median (IQR)	12.38 (3–29.09)	11.19 (2–27)		12.4 (3.0–29.1)	6.0 (1.0–17.0)	
SF-12 Physical			0.854			<0.001
Median (IQR)	48.24 (40.04–54.88)	49.52 (40.42–54.8)		48.2 (40.0–54.9)	52.5 (44.8–56.1)	
KOOS QOL			0.287			<0.001
Median (IQR)	68.75 (50–81.25)	68.75 (50–87.5)		68.8 (50.0–81.2)	75.0 (62.5–93.8)	

Supplementary Figures

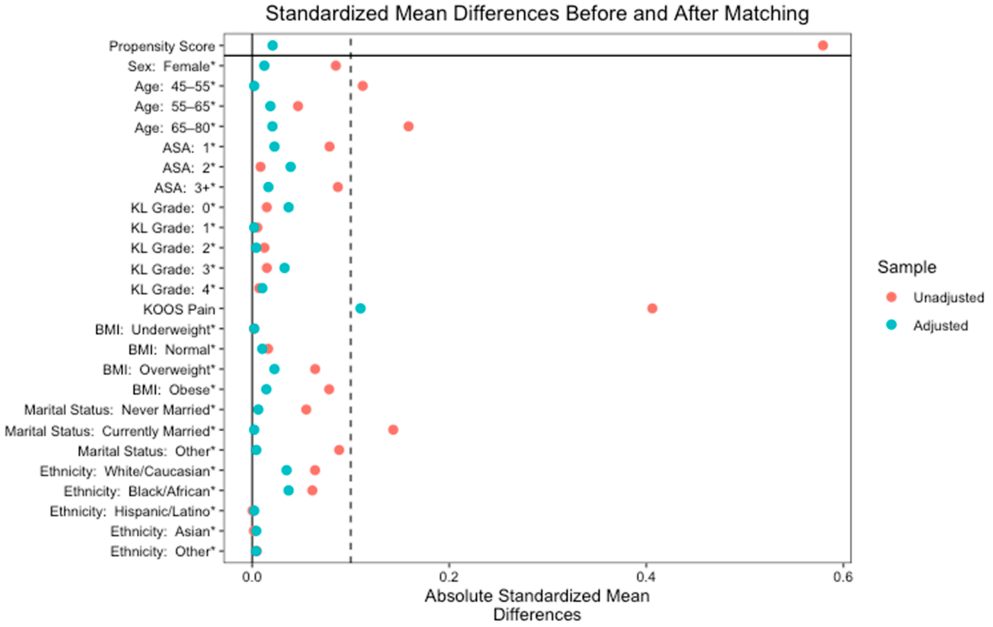

Supplementary Figure 1: Standardised Mean Differences Before and After Matching

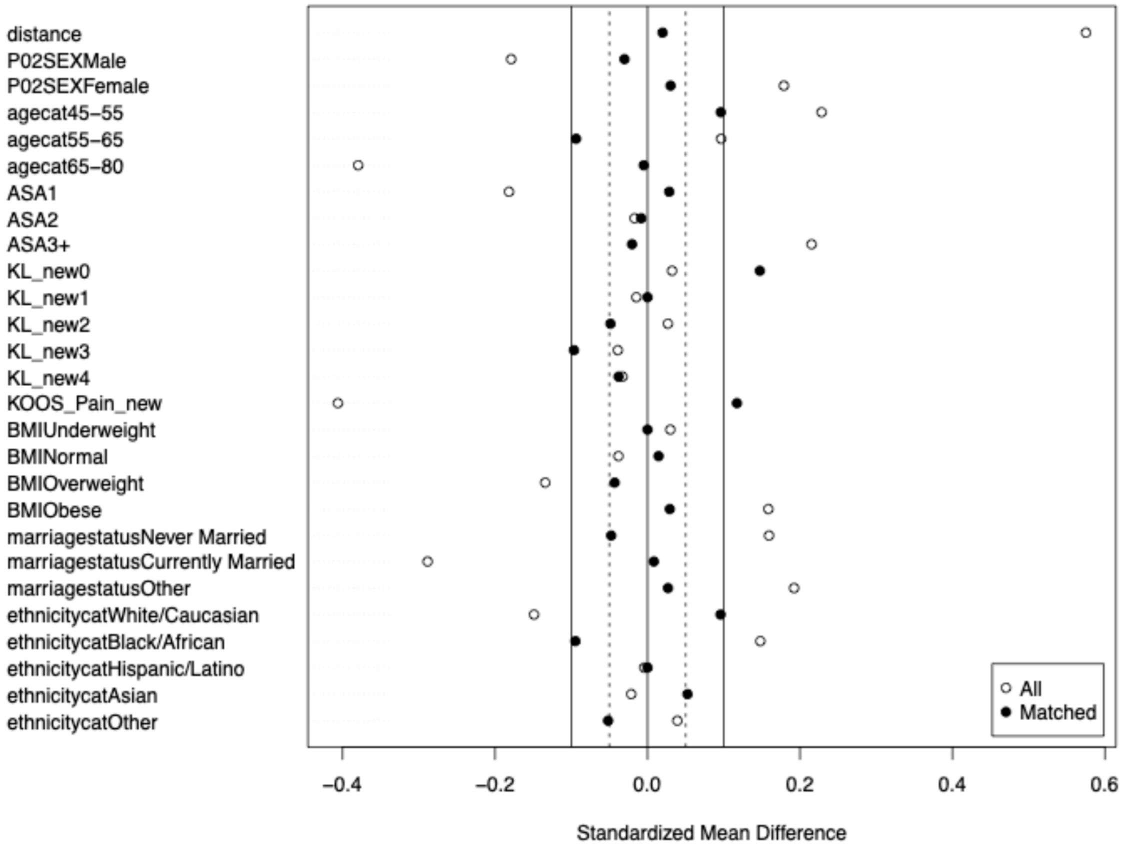

Supplementary Figure 2: Balance Plot of the Crude and Matched Data
